# Case report: Successful induction of buprenorphine/naloxone using a microdosing schedule and assertive outreach

**DOI:** 10.1186/s13722-020-0177-x

**Published:** 2020-01-15

**Authors:** Jennifer Rozylo, Keren Mitchell, Mohammadali Nikoo, S. Elise Durante, Skye P. Barbic, Daniel Lin, Steve Mathias, Pouya Azar

**Affiliations:** 1 University of British Columbia, Vancouver, BC Canada; 2 Inner City Youth Program, Providence Health Care, Vancouver, BC Canada; 3 Foundry, Providence Health Care, Vancouver, BC Canada; 4 Addiction and Concurrent Disorders Group, Institute of Mental Health, University of British Columbia, Vancouver, BC Canada; 5 St Paul’s Hospital, Vancouver, BC Canada; 6 Vancouver General Hospital, DHCC, Floor 8-2775 Laurel Street, Vancouver, BC V5Z 1M9 Canada; 7 Centre for Health Evaluation and Outcome Sciences, Vancouver, BC Canada; 8 Department of Occupational Science and Occupational Therapy, University of British Columbia, Vancouver, BC Canada

**Keywords:** Opioid use disorder, Buprenorphine/naloxone, Microdosing, Bernese method, Induction method

## Abstract

**Background:**

The requirement for moderate withdrawal prior to initiation can be a barrier to buprenorphine/naloxone induction.

**Case presentation:**

We aimed to use a microdosing regimen to initiate regular dosing of buprenorphine/naloxone in a high-risk patient with a history of failed initiations due, in part, to withdrawal symptoms. Using an assertive outreach model and a buprenorphine/naloxone microdosing schedule, we initiated treatment of an individual’s opioid use disorder. There was a successful buprenorphine/naloxone microdosing induction as the team reached a therapeutic dose of buprenorphine/naloxone. Including the induction period, the medication was used consistently for 4 weeks.

**Conclusions:**

A microdosing schedule can be used to induce a patient onto buprenorphine/naloxone with no apparent withdrawal; gradually reducing illicit substance use. This case report builds on previous literature, highlighting ways to minimize barriers to induction of buprenorphine/naloxone, using a microdosing schedule and assertive outreach. Given the safety profile of buprenorphine and its potential to be a lifesaving intervention, a larger study of microdosing is indicated.

## Background

Over the past 10 years, rates of opioid-related overdose deaths and opioid-related harms have drastically increased in British Columbia [1]. Since 2015, illicit drug use has surpassed suicide as the major cause of unnatural deaths in BC, with fentanyl-related overdoses implicated as the leading cause of illicit drug overdoses [1]. This public health crisis of historical scale has taken more lives than the HIV epidemic in the early 1990′s [2]. The latter at its peak (1995) was identified as the cause of a total of 1764 mortalities in Canada [3] compared to 4588 reported apparent opioid-related deaths in Canada in 2018 [4] when, approximately four people lost their lives to overdose every day in BC [1].

Opioid agonist treatment (OAT) has been shown to reduce morbidity and mortality among patients with opioid use disorder (OUD) [5–10]. Buprenorphine/naloxone has become the recommended first-line OAT in Canada based on its preferable safety profile and efficacy [11, 12].

Buprenorphine is a partial μ agonist, with high receptor affinity resulting in a slow dissociation from the receptor and prolonged activity. Naloxone has minimal effect when taken orally and is introduced to the formula to minimize diversion. The pharmacokinetics of buprenorphine/naloxone result in a favourable safety profile due to a ceiling effect on respiratory depression and the ability for rapid titration. Precipitated withdrawal can result if buprenorphine/naloxone is introduced in the presence of other opiates with lesser-binding affinities, such as heroin or methadone; therefore, patients are required to be in moderate withdrawal prior to induction.

Need for withdrawal prior to induction is acknowledged as a challenge for choosing OAT with buprenorphine/naloxone (BUP/NLX). This requirement mandates the patients to time their withdrawal to match an office-based appointment, and to be supervised for several hours. This is a barrier for a variety of reasons, such as lack of clinic space or staffing to monitor the induction, and also patients’ anxiety, impulsivity, work or school commitments interfering with such a long stay in the clinic. In addition to the fluctuating level of consciousness associated with high opioid use, tolerating the high cravings to use during the timed withdrawal which is required for office induction is another inherent challenge for this method. While home BUP/NLX induction strategies have offered an alternative to the need for withdrawal in clinic, there remains a selected patient population for whom the requirement for moderate withdrawal prior to initiation will remain a barrier regardless of the setting [13, 14]. Patients may also be fearful of precipitated withdrawal, which is associated with usual induction starting before adequate withdrawal. Moreover, precipitated withdrawal is perceived by some providers a barrier for adopting home induction with buprenorphine [15]. These barriers may encourage patients towards other OAT medications with less favourable safety profile such as methadone or slow-release oral morphine. Microdosing inductions can preclude the requirement for the withdrawal prior to induction and also may decrease the risk of precipitated withdrawal. Ultimately, it will also provide patients keen on starting OAT with BUP/NLX with more options.

A microdosing schedule for buprenorphine was first introduced and trialed in 2010 by Hamming et al. in Bern, Switzerland [16], followed by a more recent report of two cases of successful induction of buprenorphine/naloxone in 2016 [17]. The first case was induction of buprenorphine/naloxone using a microdosing schedule starting at 0.2 mg daily and titrated up to 12 mg daily over 9 days, with gradual reduction and eventual cessation of illicit heroin use over this time [16]. The second case was a gradual cross-titration of methadone and diacetylmorphine to buprenorphine/naloxone starting at 0.2 mg and titrated up to 24 mg over 28 days [17]. Both patients tolerated this induction without reporting the experience of precipitated withdrawal or need for withdrawal from opiates prior to induction. This method has been coined “The Bernese Method” [16]. The pharmacological hypothesis tested in the Bernese Method is that small amounts of buprenorphine doses should not precipitate opioid withdrawal, but because of its relatively long half-life, accumulates at the receptor gradually replacing the full μ-agonist (e.g. fentanyl, heroin) at the opioid receptor. This was successfully shown with these two cases presented by Hamming; however, this has not been replicated in the current practice literature [16, 17].

There has been growing interest in the Bernese Method in Vancouver, BC, Canada, as healthcare providers struggle to find ways to reduce mortality in the context of a public health emergency. To date, there has been considerable effort to engage individuals who use opioids in opioid agonist treatment, as well as to provide overdose response kits and personnel to manage acute overdoses [18]. The Bernese Method is a potential compliment to patients who want treatment with buprenorphine/naloxone, but are adverse to the traditional induction method because of the need for withdrawal and/or have difficulty attending scheduled appointments. This method has also shown promise for other indications such as pain management [19].

Apart from above-mentioned barriers, there remains other challenges for home induction with BUP/NLX. Home induction works best for patients who have stable housing, relatively good cognitive function, and are organized enough to reliably follow instructions. Unfortunately, this is not the case for most of patients who are served by the outreach programs, patients with severe opioid use disorder, high rates of cognitive impairment and major mental health illness in the most vulnerable opioid using population i.e. homeless population that can interfere with their ability to come to a clinic, tolerate withdrawal, and stay for induction and as a result precludes them often from successful home induction. Provision of microdosing within an outreach program can make buprenorphine/naloxone treatment accessible to high-risk patients who have difficulty attending office-based appointments or complying with a home-based protocol. Assertive outreach, part of this model of care, involves flexible delivery of integrated health services by an interdisciplinary team and is an established model for engaging patients with complex needs that have not been met in traditional office-based settings [20, 21]. The Inner City Youth Program (ICYP) uses this approach with high-risk youth who are living with moderate to severe mental illness and/or substance use disorders, and psychosocial and/or medical complexities. The ICYP is located at Foundry Vancouver Granville, which is a “one-stop shop” health centre in downtown Vancouver for young people aged 12–24, which includes support to family members and caregivers. Care is provided by an interdisciplinary team of peers and professionals through clinic-based and outreach services.

In February 2018, we began using a microdosing regimen to initiate regular dosing of buprenorphine/naloxone in ICYP patients with significant barriers to induction, such as developmental disabilities, homelessness, and psychosis. We used assertive outreach to identify and locate patients with OUD who were not receiving OAT and offer them buprenorphine/naloxone microdosing induction on the spot. OAT prescribers offered weekly outreach, and interdisciplinary team members provided case management and supported patients with their OAT and other related goals. Patients were linked to primary care, psychiatry, peer support and other services.

In the first 6 months of this program, 14 people, 18–25 years-old with severe disordered use of multiple substances and comorbid mental illness, and history of residential instability and poverty were engaged in care with 8 successful inductions and no instances of precipitated withdrawal. This method has also attracted growing interest among other clinics in our community. As there are limited published reports on this topic, we present a case report for discussion to contribute to the body of evidence. Specifically, we present a case of a patient successfully completing buprenorphine/naloxone induction, without reporting a period of withdrawal, using a microdosing schedule delivered via assertive outreach. Success was defined as reaching a therapeutic dose of Suboxone for a minimum of 30 consecutive days.

## Case presentation

The patient was a 55-year-old male, a parent of one of our youth outreach patients. He reported being First Nations, living in a single-room-occupancy hotel, and supported by income assistance. He had a long history of opioid and stimulant use disorders. Given the incredible urgency and need for flexibly service delivery in the context of OAT, he was taken as a patient in our youth outreach program to help family members in innovative ways. His presentation was complicated by an evolving left leg cellulitis, untreated hepatitis C, and a history of gout. At the time of initial assessment, he was not taking any medications. Our team was consulted to see him for buprenorphine/naloxone microdosing induction in his residence. Visiting the patient in his residence was used as a measure to lower the threshold for access to care and improve his engagement with the treatment.

On initial assessment, the patient self-reported injecting 200 mg of heroin daily 200 mg of crystal methamphetamine every 3 days. The actual amounts used was difficult to measure given the variability in chemical make-up and potency of heroin and other street drugs in Vancouver, including their adulteration with fentanyl and other contaminants [22, 23].

The patient had previously trialed methadone but had relapsed. He had multiple trials of traditional buprenorphine/naloxone inductions but was unable to complete them due to his intolerance of withdrawal symptoms. He had experienced at least one overdose requiring resuscitation with naloxone.

**Fig. 1:**
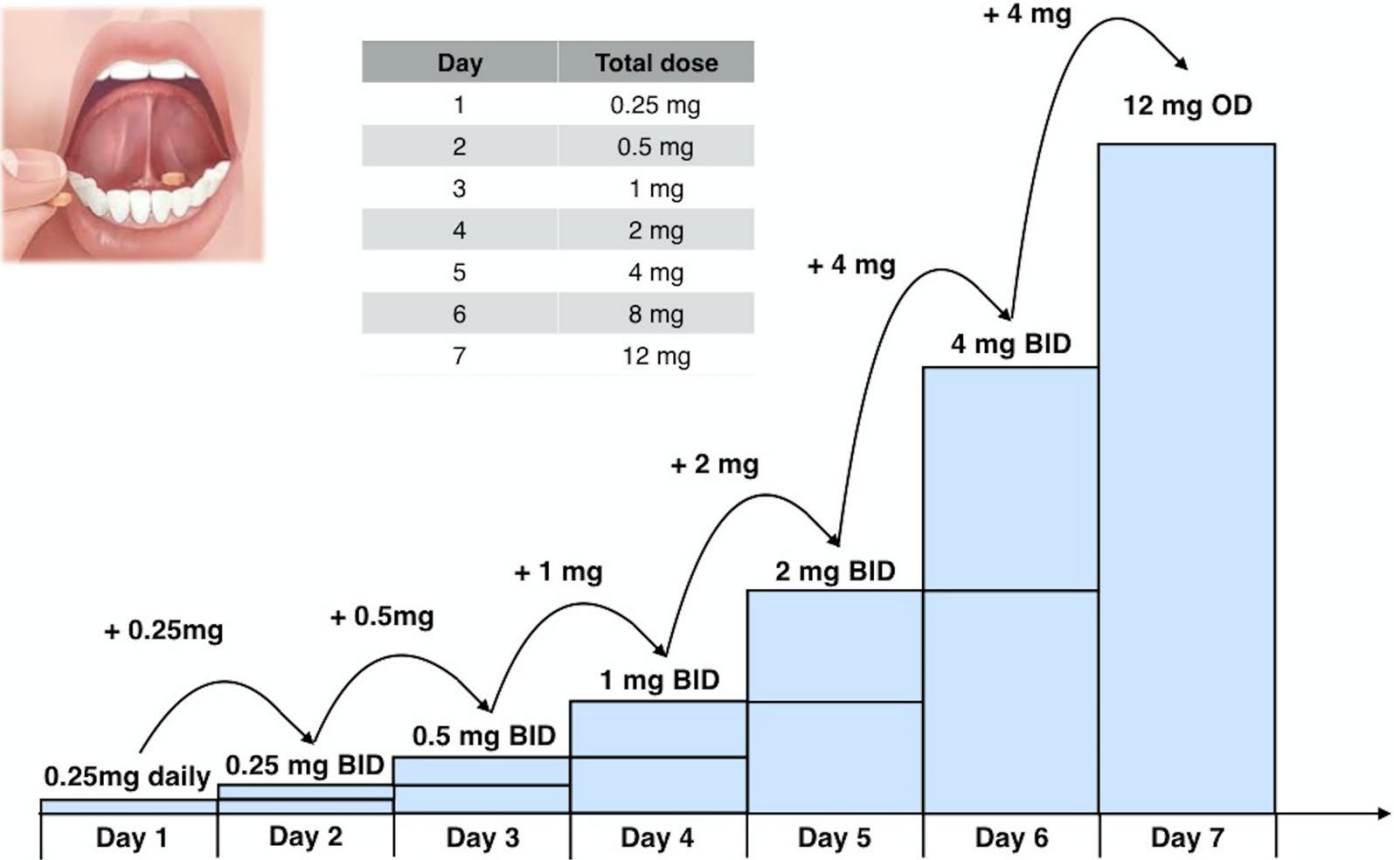
Buprenorphine/naloxone microdosing regimen (daily witness ingestion). The team partnered with a local pharmacy who were able to split that tablets for microdosing. The tablet splitting may not have been 100% accurate, however it was effective for this patient, given that the technique is to start low and gradually increase and best practice for dosing has yet to be established

Outreach visits to this patient began February 28, 2018 and he was seen four times (out of five attempted visits) over 3 weeks at his residence. The patient was prescribed a buprenorphine/naloxone microdosing regimen (see Fig. 1). The BUP/NLX tablets were split by the local pharmacy for off-label administration of small doses in microdosing protocol. Since the smallest available dose in Canada is 2 mg, it was more practical to split the tablets into 0.25 mg as opposed to 0.2 mg, which was prescribed in Bernese Method. The patient was instructed to use decreasing doses of heroin as buprenorphine/naloxone doses increased, then to stop heroin once the buprenorphine dose reached 12 mg. He completed the microdosing regimen and over the course of 7 days his dose was titrated to 12 mg daily. On day 8 the dose was increased to 16 mg daily and the patient abstained from illicit drugs. Despite the team offering to deliver the medication to the patient’s home, he chose to pick them up himself daily from the pharmacy. The outreach team supported him with reminders to pick up his medications regularly. Due to a prescription error, he missed 3 days of his buprenorphine/naloxone (March 20–23, 2018), and a relapse of heroin and crystal methamphetamine ensued. A subsequent retrial of buprenorphine/naloxone microdosing induction was prescribed, but the patient did not pick up his medications, and was difficult to find for follow up.

The patient was seen again on outreach visits to his residence and neighbourhood starting on May 14, 2018. He was contemplative about quitting heroin. He wanted to retrial the microdosing induction method. We conducted a medical history, screening for major health concerns, liver failure, other medications and allergies. Blood work from December 2017 showed normal complete blood count, electrolytes, blood urea nitrogen, and creatinine. December 2016 liver function tests were normal. A microdosing buprenorphine/naloxone regimen was re-prescribed, along with a blood requisition for updated liver function tests, human immunodeficiency virus, treponema pallidum, hepatitis B virus, complete blood count, electrolytes, blood urea nitrogen, and creatinine. Medication was dispensed daily to the patient at a pharmacy situated across the street from his residence. The prescriber and the pharmacy stayed in close phone and in-person connection regarding dosage adjustments and medication adherence.

The patient picked up and took all but two doses in the first week.

There were no symptoms of withdrawal throughout the induction. This was documented via the patients self-report and the clinicians’ overall assessment of the patient’s withdrawal symptoms as the use of standardized tools was neither feasible nor necessary given that withdrawal prior to induction is not a requirement in microdosing technique. The patient reduced illicit heroin use to 200 mg every 2 days during the week of the microdosing. The patient had mild cravings when he reached 12 mg, though decreased his use to 100 mg every three days. Follow up was challenging because the patient had difficulty keeping appointments, and we were not always able to locate him on outreach. The team looked for the patient in his home and in the neighborhood, and after several attempts assessed him on May 28, 2018. At the time, he was on 12 mg daily. He continued to self-report using heroin 500 mg every 3 days and experiencing cravings, so his dose was increased to 16 mg. He continued to stay on buprenorphine/naloxone 16 mg daily, with no further missed doses.

He was assessed again on June 11, 2018. Notably, a painful left leg cellulitis persisted, and he continued to use heroin 500 mg every 3 days when he felt leg pain. While he reported mild cravings, he reported using heroin to manage pain. The team connected him to a nearby primary care clinic for wound care and antibiotics. The dose of buprenorphine/naloxone was increased to 20 mg daily. He adhered to his agonist medication till August 27, 2018 and reported using 50 mg approximately every 4 days, which he was willing to taper. Interestingly, he reported no longer using any other illicit substances. The outreach team supported him to connect with an adult-oriented primary care and OAT clinic, and his care was transferred accordingly.

## Discussion and conclusions

We presented a case of an induction to buprenorphine/naloxone using a microdosing schedule (Bernese Method) with no apparent withdrawal. The induction was conducted successfully as part of the outreach visits to the patient’s residence in a single-room-occupancy hotel in downtown Vancouver. The outreach component, which sets this apart from the Bernese Method alone, aimed to promote adherence and minimize barriers for patients with multiple treatment failures and complex medical comorbidities. The combination of buprenorphine/naloxone safety profile, and flexible microdosing schedule are well suited to the outreach model and a complex patient population. Further studies could study the effectives of microdosing as compared to outreach component towards the effectiveness of the overall model for instance by comparing patients receiving outreach intervention with microdosing induction or regular buprenorphine dose induction. However such a study will be very challenging because traditional induction methods have been limited in their utility due to the profound executive dysfunction in this population. They are migratory and difficult to locate. They are impulsive in their substance use and struggle so much with their organization and planning that traditional induction methods are for the most part impractical. The flexibility that microdosing affords in the real-world allows induction to occur in a much more resource-efficient and achievable way.

This report shows that a microdosing schedule can be used to induce a patient onto buprenorphine/naloxone, with no apparent withdrawal, and reduced illicit substance use. Secondary benefits included increased connection with the healthcare team, and treatment of the patient’s cellulitis.

Further carefully designed research is needed to build evidence regarding the viability and efficacy of the Bernese Method in an outreach setting. The optimum dosing schedule has yet to be defined. A major limitation of this method is the continued illicit opiate use during the initial phases of the induction. The OAT may reduce or stop opioid use, but that is not the only rationale for it. Harm reduction is a pragmatic approach which focuses on immediacy of needs, patient-chosen goals, and on reducing the harms. Individuals with opioid use disorder are at extraordinarily high risk of mortality and morbidity. Opioid agonist therapy can lead to reduced opioid use, but it is also an evidence-based harm- reduction treatment in individuals who are continuing to use opioids. These include a reduction in overdoses, infectious diseases, legal problems, hospitalization and a greater stability and engagement in mental and physical health services. In addition, as in this case, usual induction is not an option for some patients and microdosing technique within the outreach program provides an alternative to ongoing use and not engaging with any sort of treatment. Hence, offering microdosing despite continued use in such cases can be considered a pragmatic harm reduction approach and may even decrease the overall risk of overdose by focussing on patients’ chosen goals and immediacy of their needs, engaging them with the treatment, and addressing often multiple concurrent illnesses such as hepatitis C, HIV, and psychosis. These yet need to be evaluated in further studies.” Also, given the high initial attrition rate of buprenorphine maintenance treatment ranging between 10 and 24% [24–26] in the first week, it is critical that novel and innovative approaches are used to overcome obstacles to initiation, e.g. requirement for withdrawal prior to induction. The Bernese Method delivered by an outreach team is a promising alternative to overcomes this obstacle while minimizing the risk for overdoses at a critical and vulnerable time. Another limitation is missing standard measurements of withdrawal and urine drug screen for illicit drug use, as the withdrawal was assessed by clinician impression and patient self-report. This could be partly explained by less than ideal setting of an outreach visit requiring optimal use of the time in a short encounter. Such measures would have allowed for a full evaluation and comparison with existing described methods. Also, the splitting of BUN/NLX tablets might not have been 100% accurate in this study; however, it was effective in that the premise is to start low and gradually increase. Best practice for dosing has yet to be established and dosing accuracy would need to be assured for future studies. Further research needs to explore feasibility of the Bernese Method in an outreach setting with a larger group of patients and warrants a comparison of the protocol versus current best practice. Further study is also needed to clarify which interventions may have assisted this patient to discontinue methamphetamine use as part of this intervention.

This case report explores a novel way to minimize barriers to induction of first-line opioid agonist treatment in Canada, buprenorphine/naloxone, by eliminating withdrawal symptoms in this phase, using a microdosing regimen based on the Bernese Method, provided as part of an outreach program.

## Data Availability

Data sharing is not applicable to this article as no datasets were generated or analysed during the current study.

## Abbreviations

OAT: opioid agonist treatment
OUD: opioid use disorder
ICY: Inner City Youth Program
ICM: intensive case management
